# Independent Association of Serum Fibroblast Growth Factor 21 Levels With Impaired Liver Enzymes in Hyperthyroid Patients

**DOI:** 10.3389/fendo.2018.00800

**Published:** 2019-01-14

**Authors:** Fangsen Xiao, Jinyang Zeng, Peiying Huang, Bing Yan, Xin Zeng, Changqin Liu, Xiulin Shi, Liying Wang, Haiqu Song, Mingzhu Lin, Shuyu Yang, Zhibin Li, Xuejun Li, Chao Liu

**Affiliations:** ^1^Endocrine and Diabetes Center, Affiliated Hospital of Integrated Traditional Chinese and Western Medicine, Nanjing University of Chinese Medicine, Nanjing, China; ^2^Department of Endocrinology and Diabetes, The First Affiliated Hospital of Xiamen University, Teaching Hospital of Fujian Medical University, Xiamen, China; ^3^Xiamen Diabetes Institute, Xiamen, China

**Keywords:** fibroblast growth factor 21, hyperthyroidism, liver enzymes, alanine transaminase, aspartate aminotransferase

## Abstract

Fibroblast growth factor 21 (FGF21) is identified as a potential biomarker for liver diseases. However, information is limited regarding serum FGF21 and impaired liver function in hyperthyroidism. We aim to determine the potential association of serum FGF21 levels with impaired liver enzymes in hyperthyroid patients. In this case-control study, 105 normal subjects and 122 overt hyperthyroid patients were included. Among them, 41 hyperthyroid patients who obtained euthyroid status after thionamide treatment received second visit. Serum FGF21 levels were determined using the ELISA method. Compared to the normal subjects, patients with hyperthyroidism had significantly elevated serum liver enzymes, including alanine transaminase (ALT) (*p* < 0.001), aspartate aminotransferase (AST) (*p* < 0.001) levels, as well as FGF21 levels (*p* < 0.001). Further analysis showed serum FGF21 (*p* < 0.05), as well as thyroid hormone (TH) free T3 (*p* < 0.05), free T4 (*p* < 0.05) levels were higher in hyperthyroid patients with impaired liver enzymes than in those with normal liver enzymes. After reversal of hyperthyroid state, elevated serum FGF21 levels in hyperthyroid patients declined significantly (*p* < 0.001), with a concomitant decrease in serum ALT (*p* < 0.001), AST (*p* < 0.001) levels. Correlation analysis showed close correlation between FGF21 and ALT (*p* < 0.002), AST (*p* < 0.012), free T3 (*p* < 0.001), free T4 (*p* < 0.001). Further logistic regression analysis revealed FGF21 is significantly associated with elevated ALT [Odds Ratio, OR 1.79, (95% confidence interval, CI), (1.30–2.47), *P* < 0.001], AST [1.59 (1.07–2.34), *p* < 0.020]. After adjustment of potential confounders, the association between FGF21 and elevated ALT remained significant [1.42 (1.01–1.99), *p* < 0.043]. In conclusion, serum FGF21 is independently associated with impaired liver enzymes in hyperthyroid patients.

## Introduction

Fibroblast growth factor 21 (FGF21), a member of FGF superfamily, emerged as a hormone involved in the regulation of glucose, lipid, and energy metabolism recently. FGF21 is abundantly expressed in the liver and secreted as a signaling protein with diverse function (1). Serum FGF21 level varies with different physiologic and pathological status, such as starvation, obesity, diabetes, and non-alcoholic fatty liver disease (NAFLD) as well. Of note, there was evidence suggesting that serum FGF21 concentrations were associated with hepatocellular function in human beings (2–4). Further study in patients with liver transplantation demonstrated that FGF21 is a sensitive predictor for severe Ischemia/reperfusion Injury (5). To date, a large body of evidence expanded the role of FGF21 as biomarker of several liver diseases. In NAFLD, plasma FGF21 levels were found to significantly and independently correlate with hepatic fat content and serve as marker of hepatic apoptosis in obese youths (2). A prospective study also showed high serum FGF21 concentration was an independent predictor of NAFLD in humans (3).

Thyroid hormone (TH) has long been recognized as a key regulator of metabolic and energy homeostasis. It regulates metabolism primarily through its actions in the liver, brown fat, and brain, etc (6). In hyperthyroidism, excess TH induces hyper-metabolic status, causes dysfunction of multiple organs. Since liver is a critical site for TH's action, hepatic dysfunction is commonly observed in hyperthyroid patients. Two case series have highlighted that prevalence of impaired liver function (ranging from 15 to 76%) in the setting of hyperthyroidism (7, 8). Recently, it was demonstrated that TH regulates adipose and hepatic FGF21 expression and serum levels in mice (9). Our previous study in hyperthyroid patients also showed serum FGF21 levels were elevated in hyperthyroidism (10). However, it is unknown whether FGF21 has any clinical implication in hyperthyroid patients. So far there is no study in human being to determine the relationship between FGF21 and liver enzyme in hyperthyroidism. Therefore, the aim of our case-control study is to establish if there is an association between FGF21 and liver enzymes in hyperthyroidism.

## Materials and Methods

### Study Subjects

This case-control study was approved by the Human Research Ethical Committee of the First Affiliated Hospital, Xiamen University (Xiamen, China) with written informed consent being obtained from all participants. The study sample comprised of 122 overt hyperthyroid patients and 105 healthy controls recruited between September, 2012 and March, 2014. All hyperthyroid patients were referred to the Department of Endocrinology and Diabetes, the First Affiliated Hospital of Xiamen University, (Xiamen, China). The inclusion criteria for hyperthyroid patients was defined as: (1) typical clinical presentation of hyperthyroidism, with elevated serum free T3 (FT3), free T4 (FT4), thyrotropin receptor autoantibody (TRAb), and reduced TSH levels; (2) All included patients did not receive any drug treatment before recruitment. Subjects with following conditions were excluded: diabetes, cancer, pregnancy, lactation, subacute thyroiditis, any confirmed liver disease such as virus hepatitis, autoimmune hepatitis, non-alcoholic fat liver disease, alcoholic liver disease, severely impaired liver function [serum alanine transaminase (ALT) and/or aspartate aminotransferase (AST) increased by more than 2-folds of the upper normal limit], abnormal kidney function (eGFR < 50 mL/min/1.73 m^2^) and infection diseases. Among hyperthyroid patients, 41 patients who accomplished euthyroid status after 3 to 6 months thionamide treatment received second visit. Venous blood was drawn during 8–9 AM after a 12 h overnight fast, and serum was separated and stored at −20°C for biochemical and FGF21 assays.

### Anthropometric and Biochemical Measurements

Body mass index (BMI) was calculated as the weight in kilograms divided by the square of the height in meters. Waist circumference was measured at the midpoint between the inferior costal margin and the superior border of the iliac crest on the mid-axillary line. HOMA-insulin resistance (HOMA-IR) was calculated by fasting serum insulin (FIns, mU/ml) ^*^fasting blood glucose (FPG, mmol/L)/22.5. Serum biochemical measurements were determined on a Hitachi 7,600 analyzer (Hitachi, Ltd., Tokyo, Japan). Elevated liver enzymes was defined as serum ALT and/or AST levels higher than upper limit of normal (40IU/l). Plasma glucose was measured using glucose oxidase method. Serum low-density lipoprotein cholesterol (LDL-C) was calculated by Friedewald's formula. Serum insulin, FT3, FT4, and TSH concentrations were measured using electrochemiluminescence immunoassay (Roche Diagnostics, Mannheim, Germany).

### FGF21 Measurement

Serum FGF21 concentrations were determined with commercially available human FGF-21 immunoassay kits (DF2100, R&D Systems, Inc., Minneapolis, MN, US) according to the manufacturer's instructions, as we described previously. This assay was verified to be highly specific for the detection of human FGF21. The detectable range of the assay was 17.0–2410.9 pg/ml. The intra- and inter-assay coefficients of variation were 5.3 and 6.7%, respectively.

### Statistical Analysis

All analyses were performed with Statistical Package for Social Sciences version 21.0 (SPSS, Chicago. IL). Normally distributed data was presented as mean ± SD. Non-normally distributed data were logarithmically transformed. Chi-square and one-way ANOVA tests were used for comparison of categorical and continuous variables, respectively. The Student's paired *t*-test was conducted for comparison of the data before and after anti-hyperthyroid treatment. Correlations between serum FGF21 levels and biochemical variables were analyzed with Pearson correlation. Multivariable logistic regression was used to establish the relationship between FGF21 and elevated liver enzymes. *P* < 0.05 was considered statistically significant.

## Results

### Baseline Serum FGF21, Liver Enzymes Levels in Study Subjects

The clinical characteristics of hyperthyroid patients and control are shown in Table 1. As expected, hyperthyroid patients have much lower serum cholesterol, LDL-c levels compared with control. Serum ALT, AST, as well as FGF21 levels are significantly higher in hyperthyroid patients as compared with age, sex-matched control group. The proportion of subject with elevated liver enzyme is also higher in hyperthyroid patients than that in control group. Subgroup analysis showed serum thyroid hormone (FT3, FT4) levels in hyperthyroid patients with elevated liver enzyme were significantly higher than those with normal liver enzyme. Interestingly, elevated serum FGF21 levels were also found in hyperthyroid patients with impaired liver enzyme.

**Table 1:** Comparison of clinical and biochemical characteristic between the study and control group.

	**Control *N* = 105**	**Hyperthyoidism**		
		**Overall *N* = 122**	**Normal liver enzymes *N* = 70**	**Impaired liver enzymes *N* = 52**
Age (year)	33.1 ± 9.0	33.7 ± 10.2	34.06 ± 10.635	33.36 ± 9.407
Gender(male/female)	33/72	32/90	50/20	40/12
BMI (kg/cm^2^)	21.18 ± 2.59	19.95 ± 2.41^a^	19.88 ± 2.33	20.22 ± 2.54
Waist circumference (cm)	74.6 ± 8.4	74.1 ± 7.5	73.98 ± 7.94	74.17 ± 7.04
SBP(mmHg)	119 ± 12	123 ± 17	123 ± 15	122 ± 19
DBP(mmHg)	75 ± 8	71 ± 10^b^	71 ± 10	71 ± 10
ALT (U/l)	17.90 ± 11.90	37.61 ± 14.89 ^a^	27.80 ± 7.24	51.57 ± 10.30^c^
AST(U/l)	20.90 ± 5.15	29.30 ± 11.31^a^	23.26 ± 5.92	37.20 ± 10.97^c^
Elevated ALT	5/105	52/122 ^a^	/	/
Elevated AST	1/105	27/122 ^a^	/	/
TBIL (μmol/L)	12.72 ± 4.74	13.58 ± 6.04	13.27 ± 6.35	13.95 ± 5.76
DBIL (μmol/L)	3.92 ± 1.23	4.55 ± 2.64	4.97 ± 2.52	4.19 ± 2.76
FPG (mmol/L)	5.37 ± 4.24	5.16 ± 0.68	5.15 ± 0.56	5.10 ± 0.74
CHO (mmol/L)	4.71 ± 0.80	3.58 ± 0.81^a^	3.57 ± 0.74	3.57 ± 0.92
TG (mmol/L)	0.89 ± 0.47	1.02 ± 0.61	0.97 ± 0.59	1.10 ± 0.64
HDL (mmol/L)	1.37 ± 0.29	1.26 ± 0.34^b^	1.25 ± 0.33	1.28 ± 0.35
LDL-c (mmol/L)	2.93 ± 0.70	1.83 ± 0.60	1.91 ± 0.56	1.78 ± 0.67
HOMR-IR^§^	1.86 (1.17–2.46)	1.42 (0.87–2.25)	1.30 (0.99–2.26)	1.46 (0.67–2.02)
Free T3 (pmol/L)^§^	4.99 (4.65–5.47)	27.76 (15.68–133.82)^a^	22.72 (15.32–107.60)	90.78 (20.51–152.64)^d^
Free T4 (pmol/L)^§^	15.00 (13.82–16.30)	65.60 (39.96–85.86)^a^	58.28 (36.84–76.50)	76.57 (51.35–99.71)^d^
TSH (mIU/L)^§^	1.78 (1.25–2.64)	0.008 (0.005–0.010)^a^	0.008 (0.006–0.011)	0.007 (0.004–0.009)
TPOAb (U/ml)^§^	5.45 (3.16–11.17)	9.06 (4.99–109.79) ^a^	11.96 (5.31–113.58)	7.21 (4.01–86.63)
TGAb (U/ml)^§^	12.14 (8.92–20.89)	27.71 (14.67–237.42)^a^	26.38 (12.00–243.91)	31.25 (14.69–129.72)
TRAb (U/ml)^§^	< 0.3	17.60 (10.33–29.94)^a^	18.05 (7.74–31.13)	17.52 (12.41–28.85)
FGF21 (pg/ml)^§^	223.1 (168.7–309.1)	282.30 (156.14–497.37)^a^	240.72 (159.00–458.70)	339.10 (156.37–797.00)^d^

§ *Analysis performed on log-transformed data*.

a *P < 0.001 compared with control group*.

b *P < 0.05 compared with control group*.

c *P < 0.001 compared with normal liver enzyme group*.

d *P < 0.05 compared with normal liver enzyme group*.

### Serum FGF21 and Liver Enzyme Levels After Anti-thyroid Drug Treatment

**Figure 1:**
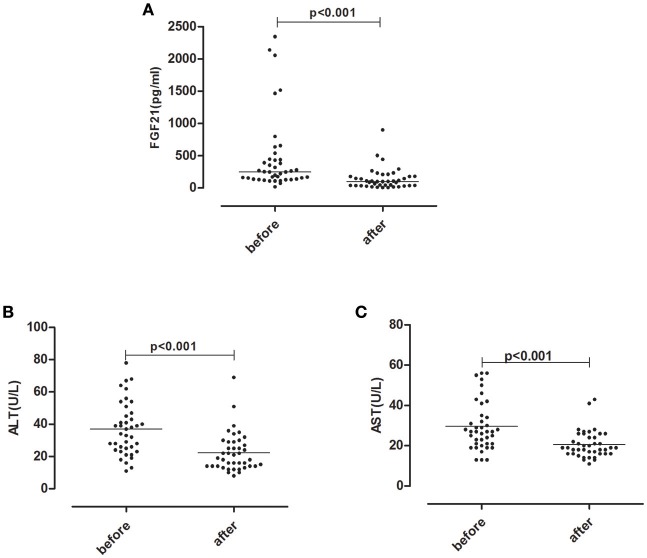
Serum FGF21 **(A)** and liver enzyme ALT **(B)**, AST **(C)** levels after anti-thyroid drug treatment in 41 hyperthyroid patients.

For 41 hyperthyroid patients received anti-thyroid drug treatment, serum FT3 and FT4 levels declined to the normal levels after treatment. Following the normalization of thyroid function, elevated serum ALT, AST levels returned to normal levels. Concomitantly, serum FGF21 levels also dropped significantly (Figure 1).

### Correlation of Serum FGF21 Levels With Liver Enzymes

**Figure 2:**
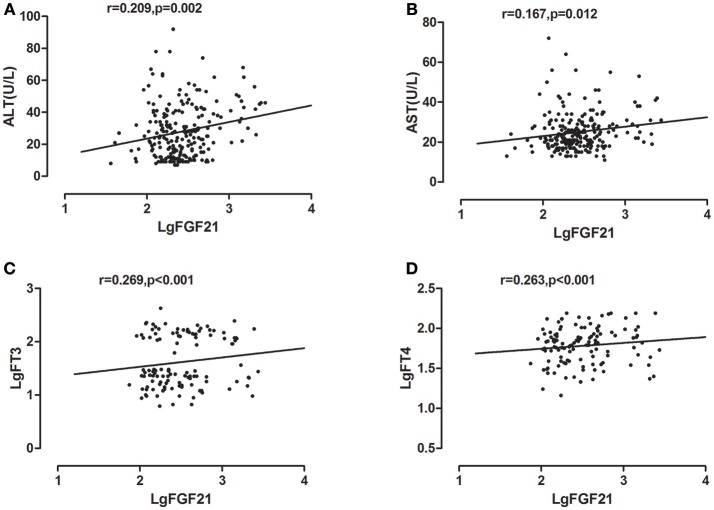
Correlation analysis between FGF21 and ALT **(A)** AST **(B)** FT3 **(C)** FT4 **(D)**.

Univariate correlations were performed to investigate factors correlated with serum FGF21, which revealed that serum ALT, AST levels were positively correlated with log- transformed serum FGF21 levels in all study subjects, with correlation coefficients of 0.209 (*p* < 0.002), 0.167 (*p* < 0.012), respectively. As we previously found, serum free T3 and free T4 level were also significantly correlated with serum FGF21 levels, with correlation coefficients of 0.269 (*p* < 0.001), 0.263 (*p* < 0.001), respectively (Figure 2).

### Association of Serum FGF21 Levels With Liver Enzymes

To further delineate the potential association between serum FGF21 and liver enzymes, we performed Logistic regression analysis across the whole cohort. As shown in Table 2, FGF21 was significantly associated with elevated ALT [OR 1.72 (95%CI 1.29–2.42), *p* < 0.001] without adjustment of potential confounders, and with elevated AST [1.59 (1.07–2.34), *p* < 0.020] as well. In model 2, with adjustment for age, gender and BMI, serum FGF21 remained significantly associated with elevated ALT [1.79 (1.30–2.47), *p* < 0.001] and AST [1.53 (1.02–2.29), *p* < 0.039]. In model 3, with further adjustment with TG, TC, and hyperthyroidism, we found serum FGF21 levels remained independently associated with elevated ALT [1.42 (1.01–1.99), *p* < 0.043], but the significant association between serum FGF21 and elevated AST disappeared [1.23 (0.82–1.84), *p* < 0.318]. In coincidence with prior reports, the final regression model also included hyperthyroidism as the independent factor associated with elevated ALT [12.82 (4.04–40.72), *p* < 0.001] and elevated AST [19.67 (2.21–175.00), *p* < 0.001].

**Table 2:** Association of serum FGF21 levels with impaired liver enzymes in the Logistic-Regression models.

**Variable**	**Odds Ratios (95% CI) for Elevated ALT**	***P*-value**	**Odds Ratios (95% CI) for Elevated AST**	***P*-value**
**MODEL 1**				
FGF21^§^	1.77 (1.29–2.42)	< 0.001	1.59 (1.07–2.34)	0.020
**MODEL 2**				
Age	1.00 (0.97–1.04)	0.957	1.04 (0.99–1.78)	0.097
Gender (Male vs. Female)	0.72 (0.34–1.53)	0.387	0.67 (0.23–1.95)	0.462
BMI	1.01 (0.89–1.15)	0.877	0.85 (0.69–1.34)	0.109
FGF21^§^	1.79 (1.30–2.47)	< 0.001	1.53 (1.02–2.29)	0.039
**MODEL 3**				
Age	0.98 (0.94–1.02)	0.279	1.02 (0.98–1.07)	0.368
Gender (Male vs. Female)	0.72 (0.30–1.72)	0.460	0.67 (0.22–2.09)	0.489
BMI	1.09 (0.94–1.27)	0.274	0.91 (0.74–1.13)	0.403
TG	2.04 (0.95–4.37)	0.068	1.14 (0.53–2.45)	0.733
TC	0.96 (0.60–1.55)	0.876	0.97 (0.52–1.79)	0.922
Hyperthyroidism (Yes vs. No)	12.82 (4.04–40.72)	< 0.001	19.67 (2.21–175.00)	0.008
FGF21^§^	1.42 (1.01–1.99)	0.043	1.23 (0.82–1.84)	0.318

§ *OR and 95%CI was expressed as per SD increase of log transferred FGF21*.

## Discussion

In the current study, we demonstrated that hyperthyroid patients had significantly higher serum FGF21 and liver enzyme levels as compared with normal controls. After reversal of the hyperthyroid state with antithyroid drug, serum liver enzyme ALT, AST levels returned to normal range which was accompanied by declined serum FGF21 levels. We also observed an independent association between FGF21 and elevated liver enzymes in hyperthyroidism. These findings provide an insight into clinical implication of FGF21 in hyperthyroid patients.

Rather than a traditional growth factor, FGF21 is considered to be a metabolic hormone. There are mounting evidences that demonstrate the role of FGF21 as a hormone regulating glucose, lipid, and energy metabolism (11). Data from liver specific knockout animals showed that circulating FGF21 mainly produced in liver (1). In humans, FGF21 is also considered nearly exclusively produced by liver (12). So FGF21 is regarded as a kind of hepatokine, namely, proteins secreted by hepatocytes that can influence metabolic processes through autocrine, paracrine and endocrine signaling (13). As a heptokine, FGF21 was widely studied in NAFLD and other liver diseases. A large body of evidence showed that Hepatic steatosis, an underlying feature of non-alcoholic fatty liver disease (NAFLD), induced changes in FGF21 secretion (14, 15). In human being, FGF21 is also identified as a biomarker of liver injury. Susan Kralisch et al. demonstrated that FGF21 serum levels are positively associated with hepatic enzymes including ALT, AST (4). In NAFLD, FGF21 was found as a biomarker of hepatic apoptosis (2). Further longevity studies also confirmed serum FGF21 as a marker of liver damage (3).

Thyroid hormone has profound and diverse effects on multiple organs. Among them, Liver serves as a key target tissue of thyroid hormone. It is well-established that hepatic dysfunction is commonly observed in patients with hyperthyroidism. The hepatic injury associated with hyperthyroidism varies from mild liver dysfunction to severe central hepatic ischemia (16). There was evidence showing that hyperthyroidism induce liver cell apoptosis in rat through the activation of death receptor-mediated pathways (17). As our previous research demonstrated serum FGF21 levels was elevated in hyperthyroid patients (10), we hypothesize that FGF21 is associated with liver injury in hyperthyroid patients. In the present study, we observed elevated serum ALT, AST, and FGF21 levels in hyperthyroid patients compared to normal control. Subgroup analysis in hyperthyroid patients demonstrated that serum FGF21 levels were significantly higher in impaired liver enzymes group than in normal liver enzyme group. Furthermore, after reversal of hyperthyroidism, liver enzyme shared the similar change trend as that of FGF21. On the other hand, there were two patients in our longitudinal cohort demonstrating distinctive changing pattern from others in serum FGF21 and ALT levels. Their serum FGF21 levels increased dramatically after antithyroid drug treatment, which was accompanied by abnormally elevated serum ALT levels (Figure S1 in Supplementary Appendix). It leads us to speculate that post-treatment serum FGF21 is likely to indicate drug-induced liver injury. Therefore, it appears there is a possible association between FGF21 and impaired liver enzymes in hyperthyroidism. Further logistic analysis clearly showed that serum FGF21 was independently associated with liver enzymes in hyperthyroid patients, with adjustment of potential confounders. Hence FGF21 may be a potential biomarker for liver function impairment or drug-induced liver injury in hyperthyroidism, which needed to be further investigated in the future.

Our study has several limitations. First, the sample size was relatively small, the population of subclinical hyperthyroidism was not included, which hampered the power of our study. Second, we didn't measure cytokeratin 18, which is a biomarker of liver cell apoptosis. Third, due to its cross-sectional nature, we should bear in mind that it is impossible to determine a cause and effect relationship between FGF21 and liver enzymes in our study.

In conclusion, compared with normal control, circulating FGF21 levels are significantly increased in hyperthyroid patients and are independently associated with impaired liver enzymes. Our study provides for first time the clinical evidence for relevance of FGF21 and liver enzymes in hyperthyroidism.

## Ethics Statement

All procedures performed in studies involving human participants were in accordance with the ethical standards of the institutional and/or national research committee and with the 1964 Helsinki declaration and its later amendments or comparable ethical standards.

## Author Contributions

FX, ZL, XL, and ChaoL designed the research. FX, JZ, PH, BY, ChaoL, XS, and HS conducted the research. FX, XZ, ChanL, LW and ZL analyzed the samples and data. FX, ML, SY, and XL wrote the article. FX, XL, and ChaoL had primary responsibility for final content. All authors read and approved the final manuscript.

### Conflict of Interest Statement

The authors declare that the research was conducted in the absence of any commercial or financial relationships that could be construed as a potential conflict of interest.

## Abbreviations

BMI: body mass index
SBp: systolic blood pressure
DBp: diastolic blood pressure
ALT: Alanine transaminase
AST: glutamic oxalacetic transaminase
TBIL: total bilirubin
DBIL: direct bilirubin
FPG: fast plasma glucose
CHO: cholesterol
TG: triglyceride
HDL: high density lipoprotein
LDL-c: low density lipoprotein cholesterol
FIns: fast serum insulin
HOMA-IR: homeostasis model assessment method insulin resistance
FT3: free tri-iodothyronine
FT4: free thyroxin
TSH: thyroid stimulating hormone
TPOAb: thyroid peroxide antibody
TGAb: thyroid globulin antibody
TRAb: thyrotrophin receptor antibody.
