# Supplementary material for: Independent Association of Serum Fibroblast Growth Factor 21 Levels With Impaired Liver Enzymes in Hyperthyroid Patients
Source: Front Endocrinol (Lausanne). 2019 Jan 14;9:800. doi: 10.3389/fendo.2018.00800 (PMC6339876; doi:10.3389/fendo.2018.00800)
Supplement: Supplementary file 1 [file Data_Sheet_1.PDF]

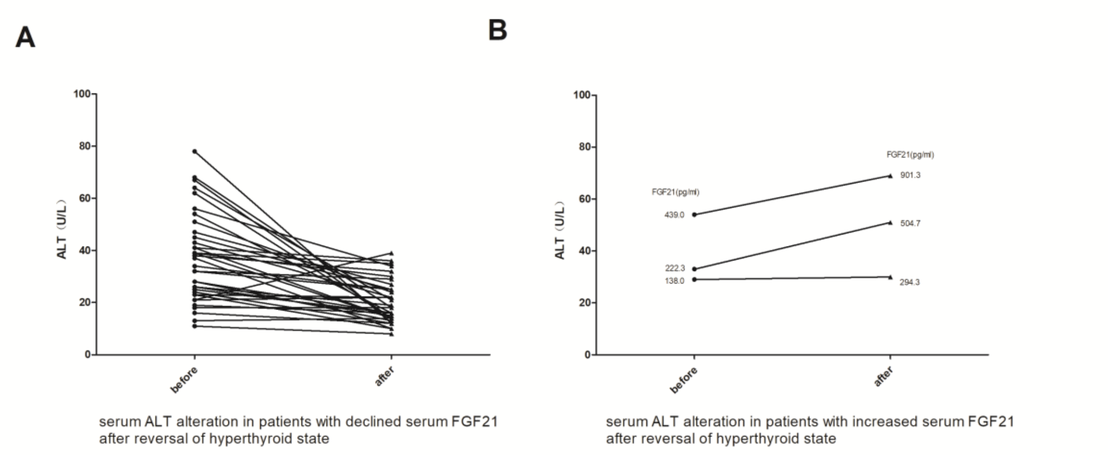

FigS1. Serum ALT alteration in patients with declined (A) and increased(B) serum FGF21 levels after reversal of hyperthyroid state. Individual FGF21 value was marked respectively besides the corresponding ALT symbol in Figure B.
